# Severe Blood–Brain Barrier Disruption in Cardioembolic Stroke

**DOI:** 10.3389/fneur.2018.00055

**Published:** 2018-02-08

**Authors:** Chang Liu, Feina Shi, Zhicai Chen, Shenqiang Yan, Xinfa Ding, Min Lou

**Affiliations:** ^1^Department of Neurology, School of Medicine, The 2nd Affiliated Hospital of Zhejiang University, Hangzhou, China; ^2^Department of Radiology, School of Medicine, The 2nd Affiliated Hospital of Zhejiang University, Hangzhou, China

**Keywords:** acute ischemic stroke, blood–brain barrier, cardioembolism, CT perfusion, stroke subtypes

## Abstract

**Background:**

Previous studies demonstrated that cardioembolism (CE) was prone to develop hemorrhagic transformation (HT), whereas hyper-permeability of blood–brain barrier (BBB) might be one reason for the development of HT. We, thus, aimed to investigate whether the BBB permeability (BBBP) was higher in CE stroke than other stroke subtypes in acute ischemic stroke (AIS) patients.

**Methods:**

This study was a retrospective review of prospectively collected clinical and imaging database of AIS patients who underwent CT perfusion. Hypoperfusion was defined as Tmax >6 s. The average relative permeability-surface area product (rPS), reflecting the BBBP, was calculated within the hypoperfusion region (rPS_hypo_). CE was diagnosed according to the international Trial of Org 10172 in Acute Stroke Treatment criteria. Receiver operating characteristics (ROC) curve analysis was used to determine predictive value of rPS_hypo_ for CE. Logistic regression was used to identify independent predictors for CE.

**Results:**

A total of 187 patients were included in the final analysis [median age, 73 (61–80) years; 75 (40.1%) females; median baseline National Institutes of Health Stroke Scale score, 12 (7–16)]. Median rPS_hypo_ was 65.5 (35.8–110.1)%. Ninety-seven (51.9%) patients were diagnosed as CE. ROC analysis revealed that the optimal rPS_hypo_ threshold for CE was 86.71%. The value of rPS_hypo_ and the rate of rPS_hypo_>86.71% were significantly higher in patients with CE than other stroke subtypes (*p* < 0.05), after adjusting for the potential confounds.

**Conclusion:**

The extent of BBB disruption is more severe in CE stroke than other stroke subtypes during the hyperacute stage.

## Introduction

Cardioembolism (CE) is one of the major causes of ischemic stroke and its outcome is usually worse than other stroke subtypes (1). Studies have shown hemorrhagic transformation (HT), a potentially life-threatening complication of stroke, was more common in CE (2, 3). Considering that the underlying mechanism of HT was the hyper-permeability, even damage of blood–brain barrier (BBB) (4, 5), we hypothesized that CE might associate with higher BBB permeability (BBBP) than other stroke subtypes.

CT perfusion (CTP) is a modern dynamic imaging technique that can evaluate BBBP by generating permeability-surface area product (PS) maps. Most studies focused on the relationship between BBBP and HT, while few explored the association between BBBP and stroke subtypes. Therefore, we aimed to assess the association between BBBP and CE, especially BBBP in the hypoperfusion region where most HT occurs.

## Materials and Methods

### Patients

We reviewed our consecutively collected patients with acute ischemic stroke who received intravenous thrombolysis (IVT) between July 2011 to January 2016. We then enrolled patients who (1) underwent CTP before IVT suggesting acute anterior circulation stroke; (2) baseline hypoperfusion (defined as Tmax >6 s) volume >10 ml. Exclusion criteria: (1) image quality was insufficient for analysis; (2) bilateral ischemic stroke. Intravenous alteplase was administered according to the international guidelines (0.9 mg/kg, 90 mg dose at maximum, 10% in a bolus in 1 min with the remaining dose in a 60-min infusion) (6).

We retrieved demographic, clinical, and laboratory data including age, sex, smoking history, comorbid conditions, such as history of hypertension, diabetes, hyperlipidemia, and atrial fibrillation; National Institutes of Health Stroke Scale (NIHSS) score, and onset-to-imaging time (OIT).

Stroke etiology was classified according to the Trial of Org 10172 in Acute Stroke Treatment (TOAST) criteria: large-artery atherosclerosis (LAA), cardioembolism (CE), small-artery occlusion (SAO), acute stroke of other determined etiology (ODE), and stroke of undetermined etiology (UDE) (7). Patients were dichotomized into CE group versus non-CE group.

### Imaging Protocols

CT perfusion was performed on a 2 × 64-slice CT scanner (SOMATOM Definition Flash; Siemens Healthcare Sector, Forchheim, Germany), including non-enhanced head CT scan (120 kV, 320 mA, effective dose = 2.19 mSv, contiguous 5 mm axial slices), and volume perfusion CT (VPCT) (4 s delay after start of contrast medium injection, 74.5 s total imaging duration, 80 kV, 120 mA, effective dose = 3.68 mSv). VPCT consisted of 26 consecutive spiral acquisitions of the brain from the base to the top of the skull, and were divided into four parts: (1) 2 scans with 3 s cycle time; (2) 15 scans with 1.5 s cycle time; (3) 4 scans with 3 s cycle time; and (4) 5 scans with 6 s cycle time. A 60-ml bolus of non-ionic contrast medium (Iopamidol; 370 mg/ml; Bracco Sine, Shanghai, China) was used at a flow rate of 6 ml/s, followed by a 20 ml saline chaser at 6 ml/s.

### Image Analysis

Cerebral blood flow (CBF) and Tmax were automatically calculated from CTP data with commercially available software (MIStar, Apollo Medical Imaging Technology, Melbourne, VIC, Australia). CTP-derived PS color maps were also automatically generated by MIStar permeability model based on the adiabatic approximation of tissue homogeneity model (8–10). A threshold of Tmax >6 s was used for volumetric measurement of baseline hypoperfusion areas (11). Baseline relative CBF <30% was used for calculating infarct volume (12). The baseline mismatch ratio was defined as the volume of hypoperfusion divided by the corresponding infarct volume (13).

Baseline hypoperfusion regions in the ischemic hemisphere were automatically outlined by MIStar on Tmax maps as regions of interest (ROI)s in each slice separately. These ROIs were manually checked and corrected by an experienced neuro-radiologist (Xinfa Ding) and two trained observers (Chang Liu and Feina Shi) who were blinded to other study data. We then superimposed these ROIs on the PS maps and CBF maps by each slice (thickness = 10 mm), respectively, to calculate mean PS and CBF values within all ROIs. The mean PS and CBF were measured by squared voxels. PS represents the rate of iodinated contrast extravasation from the intravascular to the extravascular space through a damaged BBB, and is influenced by CBF at the condition of ischemia. Therefore, it is more appropriate to use relative permeability-surface area product (rPS, rPS = PS/CBF × 100%) to represent BBBP in stroke patients (14, 15). The CBF and rPS of the hypoperfusion region were represented as CBF_hypo_ and rPS_hypo_, respectively.

Hemorrhagic transformation was defined as presence of hemorrhagic infarction (HI) or parenchymal hematoma (PH) at 24 h after treatment according to European Cooperative Acute Stroke Study criteria (16).

### Statistical Analysis

Median (25th–75th percentile) for non-normal distributed continuous variables and frequency (percentage) for categorical variables were used. Receiver operating characteristics (ROC) curve analysis was used to determine predictive value of rPS_hypo_ for CE. Chi-square test was used to compare the dichotomous variables between two groups, while independent samples Mann–Whitney *U* test was used for non-normal distributed continuous variables. We performed multivariable logistic regression analysis to assess independent predictors of CE. Results are reported as odds ratios with 95% confidence intervals (CIs). A significance level of 0.05 was used to assess statistical difference. All statistical analyses were performed using SPSS Version 19.0 (IBM, Armonk, NY, USA).

## Results

Of 230 patients who met inclusion criteria, 43 patients were excluded [poor image quality due to excessive motion during imaging and trouble in contrast injection which affected measurement of PS (*n* = 24) and diagnosis of bilateral ischemic stroke (*n* = 19)]. A total of 187 remaining patients were included for the final analysis, of which 158 patients underwent IVT alone and 29 patients had IVT combined with endovascular therapy. The median age was 73 (61–80) years, and 75 (40.1%) were female. Median baseline NIHSS score was 12 (7–16), and median OIT was 163 (96–240) min. rPS_hypo_ ranged from 4.2–321.4% and its median value was 65.5 (35.8–110.1)%. Demographic and clinical characteristics of patients included in and excluded from the current study were comparable (Table S1 in Supplementary Material).

According to TOAST criteria, 97 (51.9%) patients were diagnosed as CE, of whom 16 patients did not have atrial fibrillation: 2 had mechanical prosthetic valves, 3 had dilated cardiomyopathy, 1 had mitral annulus calcification, 9 had congestive heart failure, 1 had myocardial infarction. Numbers of patients diagnosed as LAA, SAO, ODE, and UDE were 43 (23.0%), 0. 1 (0.5%) and 46 (24.6%), respectively. 14 patients were diagnosed as non-CE-presenting atrial fibrillation, among whom 11 patients were classified as A1-S0-C1-O0-D0, and 3 patients were classified as A1-S3-C1-O0-D0 according to ASCOD classification (17).

Receiver operating characteristics analysis revealed an optimal rPS_hypo_ threshold for CE of 86.71%, with an area under the curve of 0.642 (95% CI 0.563–0.721, *p* = 0.001), and this yielded a sensitivity of 44.3% and a specificity of 80.0%. As showed in Table 1, patients in CE group were older, and had higher female predominance and lower rate of current smokers than non-CE group (all *p* < 0.05). The platelet counts and leukocyte counts were lower in CE group (both *p* < 0.05). Baseline NIHSS, infarct volume and hypoperfusion volume were comparable between groups, while baseline mismatch ratio was significant lower in CE group (*p* = 0.033). Compared with non-CE group, CE group had lower CBF_hypo_ (*p* = 0.032) and greater rPS_hypo_ (*p* = 0.001). CE group had higher rate of HT than non-CE group (*p* = 0.038), while the rates of HI and PH were comparable.

**Table 1:** Univariate comparison between CE group and non-CE group.

	CE (*n* = 97)	Non-CE (*n* = 90)	*p*-Value
Age, years	75 (66–80)	69 (57–79)	0.008
Female, *n* (%)	50 (51.5)	25 (27.8)	0.001
Smoking, n (%)	24 (24.7)	40 (44.4)	0.005
Hypertension, *n* (%)	71 (73.2)	54 (60.0)	0.055
Diabetes, *n* (%)	19 (19.6)	15 (16.7)	0.605
Hyperlipidemia, *n* (%)	39 (40.2)	41 (45.6)	0.460
Atrial fibrillation, *n* (%)^a^	81 (83.5)	14 (15.6)	<0.001
Serum glucose, mg/mL	6.9 (6.1–8.2)	6.8 (5.9–8.2)	0.460
Platelet counts, ×10^9^/L	167 (135–199)	196 (167–229)	<0.001
Leukocyte counts, ×10^9^/L	7.0 (5.8–8.7)	8.7 (6.9–11.4)	<0.001
Baseline NIHSS score	12 (8–16)	12 (5–16)	0.181
Baseline infarct volume, mL	37.2 (19.6–65.4)	24.5 (11.9–62.6)	0.122
Baseline hypoperfusion volume, mL	87.2 (48.2–134.2)	84.3 (32.6–154.7)	0.794
Baseline mismatch ratio	2.3 (1.7–3.2)	2.5 (1.9–3.9)	0.033
CBF_hypo_, ml/min/100 g	6.22 (4.61–8.37)	7.21 (5.33–9.44)	0.032
rPS_hypo_, %	75.90 (48.39–126.03)	55.60 (26.89–82.57)	0.001
rPS_hypo_ > 86.71%, *n* (%)	43 (44.3)	18 (20)	0.001
HT, *n* (%)	46 (47.4)	29 (32.2)	0.038
HI, *n* (%)	35 (36.1)	21 (23.3)	0.078
PH, *n* (%)	11 (11.3)	8 (8.9)	0.634

*CBF_hypo_, cerebral blood flow in hypoperfusion region; CE, cardioembolism; HI, hemorrhagic infarction; HT, hemorrhagic transformation; NIHSS, national institute of health stroke scale; PH, parenchymal hematoma; rPS_hypo_, rPS in hypoperfusion region*.

*^a^Atrial fibrillation was not included in the binary logistic regression model*.

Since atrial fibrillation was highly correlated with CE, it was not selected for adjustment in the binary logistic regression. Table 2 shows the results of multivariable logistic regression analysis and demonstrated that only female, platelet counts, rPS_hypo_ as well as rPS_hypo_ > 86.71% were independently associated with CE (all *p* < 0.05).

**Table 2:** Multivariable logistic regression analysis for cardioembolism.

	Odds ratios	95% confidence interval	*p*-Value
**Model 1**			
Age, years	1.015	0.988–1.043	0.290
Female	4.026	1.682–9.637	0.002
Smoking	0.971	0.419–2.253	0.946
Platelet counts, ×10^9^/L	0.988	0.981–0.995	0.001
Leukocyte counts, ×10^9^/L	0.894	0.779–1.025	0.109
Baseline mismatch ratio	1.017	0.932–1.109	0.710
CBF_hypo_, ml/min/100 g	0.949	0.825–1.091	0.462
rPS_hypo_, %	1.008	1.001–1.014	0.029

**Model 2**			
Age, years	1.013	0.985–1.041	0.369
Female	4.056	1.683–9.775	0.002
Smoking	1.043	0.444–2.450	0.924
Platelet counts, ×10^9^/L	0.987	0.979–0.994	0.001
Leukocyte counts, ×10^9^/L	0.889	0.773–1.021	0.095
Baseline mismatch ratio	1.026	0.939–1.211	0.569
CBF_hypo_, ml/min/100 g	0.964	0.840–1.106	0.604
rPS_hypo_ > 86.71%	3.754	1.610–8.753	0.002

*CBF_hypo_, cerebral blood flow in hypoperfusion region; rPS_hypo_, rPS in hypoperfusion region*.

## Discussion

To the best of our knowledge, this is the first study to evaluate the association between quantitatively assessed BBBP and stroke subtypes at hyperacute stage. We found that patients with CE had significantly higher baseline rPS_hypo_ than those with other stroke subtypes.

Previously, a qualitative study found CE was independently associated with BBB disruption, which was defined as the presence of an intraparenchymal hyperdense area exhibited on non-enhanced CT immediately after endovascular therapy (18). In our study, we quantitatively assessed BBBP on PS maps generated from pretreatment CTP. And we found that quantitative BBBP on pretreatment CTP was also associated with stroke subtype.

BBB permeability is elevated in hypoperfusion region as a result of loss of CBF, oxygen, and nutrients (19), while collateral circulation is thought to ameliorate the consequence of the above events (20). Previous study found that less elevated BBBP was associated with more than 50% collateral filling (21). Cardioembolic occlusion occurs abruptly, and its collateral circulation is thought to be insufficient, leading to more severe hypoperfusion, and therefore higher BBBP compared with LAA (22, 23). Interestingly, in the current study, there was no significant difference in either infarct volume or hypoperfusion volume between CE group and non-CE group. It may be partially due to the exclusion of patients with hypoperfusion volume ≤10 ml. We think the sudden occlusion of artery and lack of collateral circulation may be the major reason of high BBBP in CE. Besides, Olivot et al. found that endogenous tissue-type plasminogen activator (t-PA) was increased in CE (24). Previous study also found patients with atrial fibrillation, regardless of their history of ischemic stroke, had higher endogenous t-PA levels than patients in sinus rhythm (25). Set aside its thrombolytic effect, t-PA could be toxic to ischemic brain tissue, including BBB, through t-PA-dependent pathways and plasmin-dependent pathways (26, 27). This can be another evidence that links BBB disruption and CE. Moreover, previous studies revealed that C-reactive protein (CRP) was higher in patients with CE (28, 29). It has been proved that CRP could cause endothelial cells contraction, which went along with disruption of tight junction molecules and finally resulting in BBB disruption (30). Matrix metalloproteinase 9, which could disrupt BBB through degradation of extracellular matrix components of the basal lamina, was also increased after CE stroke (31). All of these pieces of evidence (including ours) indicate that CE might be associated with increased BBBP.

Our finding about the hyper-permeability of BBB in CE patients may suggest that the application of BBB protecting drugs is helpful especially for CE patients in the future. Interestingly, a recent study found that BBB disruption was reversible after reperfusion (32), although high baseline BBBP might lead to HT and malignant edema (33). Moreover, the increase of reperfusion rate was positively associated with the decrease of BBBP (32). Therefore, reperfusion therapy would still be a good choice for CE patients at acute stage. As we mentioned before, CE patients had more endogenous t-PA that might be harmful to BBB. Nevertheless, considering the benefit of recanalization, intravenous recombinant t-PA should still be the first aid for CE within time window according to the guideline.

Our study had several limitations. First, the retrospective design might have a potential risk of selection bias. All patients in this study received IVT. Because treatment should not affect pretreatment BBBP as well as stroke subtype, the results should not be affected by the selected patients. About 10% patients in our study were excluded because of inadequate image quality for the assessment of PS. The measurement of PS is based on arterial input function (AIF) curve and venous output function (OVF) curve. Most defective AIF and OVF curve are due to excessive motion during imaging and trouble in contrast injection, which are unavoidable in practice. Second, patients with hypoperfusion volume less than 10 ml were not included in this study. One reason is for its low signal-to-noise ratio; another is that when drawing ROIs in such small areas, relatively larger manual measurement error may be introduced. Third, the CTP measurement technique used in this study was first-pass with a short acquisition time (74.5 s). Previous studies suggested that it was better to use BBBP estimated from the delayed phase (34, 35). However, there was a linear relationship between BBBP measured from first-pass and delayed phase (36). In addition, the delayed phase extends the acquisition time to at least 210 s, which may further increase the radiation dose and risk of motion artifacts. Our sequence is much more practical and generalizable to everyday clinical practice. Fourth, there were 14 patients had atrial fibrillation in non-CE group and all of them had stenosis of ipsilateral major cerebral artery over 50%. We could not exclude the role of atrial fibrillation in infarct among these patients, which might lead to the underestimate of the difference of BBBP between CE group and non-CE group.

In conclusion, the current study demonstrated that BBBP in the hypoperfusion region was higher in CE patients than other stroke subtypes, indicating severe BBB disruption in cardioembolic stroke. Further prospective studies are needed to confirm this finding.

## Ethics Statement

All subjects had given written informed consent prior to the study, and the protocols had been approved by the human ethics committee of the Second Affiliated Hospital of Zhejiang University School of Medicine. All clinical investigations have been conducted according to the principles expressed in the Declaration of Helsinki.

## Author Contributions

CL drafted and revised the manuscript, participated in study concept and design, conducted the statistical analyses, analyzed, and interpreted the data. ML participated in study concept and design, data interpretation, and made a major contribution in revising the manuscript. FS, ZC, and SY participated in the study design and made contribution in revising the manuscript. CL, FS, and XD participated in the data acquisition and analysis.

## Conflict of Interest Statement

The authors declare that the research was conducted in the absence of any commercial or financial relationships that could be construed as a potential conflict of interest.
