# Supplementary material for: Severe Blood–Brain Barrier Disruption in Cardioembolic Stroke
Source: Front Neurol. 2018 Feb 8;9:55. doi: 10.3389/fneur.2018.00055 (PMC5809413; doi:10.3389/fneur.2018.00055)
Supplement: Supplementary file 1 [file table_1.docx]

Supplementary Table 1. Comparison between patients included in and excluded from the current study

|  | Included patients (n=187) | Excluded patients (n=43) | *p* value |
| --- | --- | --- | --- |
| Age, y | 73(61-80) | 69(60-80) | 0.532 |
| Female, n (%) | 75(40.1) | 16(37.2) | 0.863 |
| Onset-to-needle time, min | 197(135-272) | 192(139-268) | 0.712 |
| Risk factors |  |  |  |
| Smoking, n (%) | 64(34.2) | 16(37.2) | 0.725 |
| Hypertension, n (%) | 125(66.8) | 34(79.1) | 0.144 |
| Diabetes, n (%) | 34(18.2) | 9(20.9) | 0.668 |
| Hyperlipidemia, n (%) | 80(42.8) | 13(30.2) | 0.168 |
| Atrial fibrillation, n (%) | 95(50.8) | 24(55.8) | 0.613 |
| Serum glucose, mg/mL | 6.9(6.1-8.2) | 6.8(5.9-8.4) | 0.817 |
| Platelet counts, ×10^9^/L | 180(147-214) | 175(136-204) | 0.114 |
| Leukocyte counts, ×10^9^/L | 7.9(6.3-10.1) | 7.7(6.3-10.7) | 0.762 |
| Baseline NIHSS score | 12(7-16) | 12(7-19) | 0.707 |
| Baseline infarct volume, mL | 32.0(13.8-63.9) | 36.5(7.8-92.8) | 0.911 |
| Baseline hypoperfusion volume, mL | 86.6(43.9-139.8) | 113.7(40.8-201.5) | 0.183 |
| Hemorrhage transformation, n (%) | 75(40.1) | 18(41.9) | 0.864 |
| 90-day mRS | 3(1-4) | 4(1-5) | 0.069 |
| 90-day mRS > 2, n (%) | 98(52.4) | 24(55.8) | 0.737 |
| Cardioembolic stroke, n (%) | 97(51.9) | 22(51.2) | 0.933 |

NIHSS: national institute of health stroke scale; mRS: modified Rankin scale.

Supplementary Table 2. Collinearity statistics

| Variables | Tolerance | Variance inflation factor |
| --- | --- | --- |
| Age, y | 0.863 | 1.159 |
| Female | 0.639 | 1.564 |
| Smoking | 0.657 | 1.522 |
| Platelet counts, ×10^9^/L | 0.792 | 1.263 |
| Leukocyte counts, ×10^9^/L | 0.765 | 1.308 |
| Baseline mismatch ratio | 0.936 | 1.068 |
| CBF_hypo_, ml/min/100g | 0.688 | 1.452 |
| rPS_hypo_，% | 0.703 | 1.423 |

CBF_hypo_: cerebral blood flow in hypoperfusion region; rPS_hypo_: rPS in hypoperfusion region.

Supplementary Table 3. Collinearity diagnostics

| Dimension | Eigenvalue | Condition Index | Variance Proportions | | | | | | | | |
| --- | --- | --- | --- | --- | --- | --- | --- | --- | --- | --- | --- |
|  |  |  | (Constant) | age | Female | Smoking | Platelet counts | Leukocyte counts | Baseline mismatch ratio | CBF_hypo_ | rPS_hypo_ |
| 1 | 6.518 | 1.000 | 0.00 | 0.00 | 0.00 | 0.00 | 0.00 | 0.00 | 0.01 | 0.00 | 0.00 |
| 2 | 1.009 | 2.542 | 0.00 | 0.00 | 0.16 | 0.22 | 0.00 | 0.00 | 0.03 | 0.00 | 0.01 |
| 3 | 0.607 | 3.277 | 0.00 | 0.00 | 0.03 | 0.02 | 0.00 | 0.00 | 0.64 | 0.00 | 0.10 |
| 4 | 0.403 | 4.020 | 0.00 | 0.00 | 0.05 | 0.05 | 0.00 | 0.00 | 0.27 | 0.03 | 0.39 |
| 5 | 0.232 | 5.306 | 0.00 | 0.00 | 0.67 | 0.66 | 0.00 | 0.02 | 0.03 | 0.04 | 0.01 |
| 6 | 0.107 | 7.789 | 0.00 | 0.03 | 0.00 | 0.02 | 0.05 | 0.43 | 0.02 | 0.23 | 0.02 |
| 7 | 0.058 | 10.577 | 0.01 | 0.13 | 0.06 | 0.00 | 0.24 | 0.18 | 0.00 | 0.52 | 0.33 |
| 8 | 0.053 | 11.064 | 0.02 | 0.11 | 0.02 | 0.01 | 0.69 | 0.18 | 0.00 | 0.13 | 0.12 |
| 9 | 0.012 | 23.326 | 0.96 | 0.73 | 0.00 | 0.01 | 0.01 | 0.18 | 0.00 | 0.05 | 0.02 |

CBF_hypo_: cerebral blood flow in hypoperfusion region; rPS_hypo_: rPS in hypoperfusion region.

Supplementary Table 4. Univariate comparison between atrial fibrillation (AF) group and non-AF group

|  | AF (n=95) | Non-AF (n=92) | *p* value |
| --- | --- | --- | --- |
| Age, y | 75(67-80) | 67(57-79) | 0.001 |
| Female, n (%) | 51(53.7) | 24(26.1) | <0.001 |
| Risk factors |  |  |  |
| Smoking, n (%) | 24(25.3) | 40(43.5) | 0.009 |
| Hypertension, n (%) | 69(72.6) | 56(60.9) | 0.088 |
| Diabetes, n (%) | 15(15.8) | 19(20.7) | 0.389 |
| Hyperlipidemia, n (%) | 35(36.8) | 45(48.9) | 0.095 |
| Serum glucose, mg/mL | 6.9(6.1-8.1) | 6.9(5.9-8.3) | 0.728 |
| Platelet counts, ×10^9^/L | 169(130-201) | 194(163-231) | <0.001 |
| Leukocyte counts, ×10^9^/L | 7.1(5.9-8.3) | 8.9(6.8-11.5) | <0.001 |
| Baseline NIHSS score | 12(8-17) | 12(6-16) | 0.102 |
| Baseline infarct volume, mL | 32.4(13.6-64.7) | 32.0(14.1-63.3) | 0.915 |
| Baseline hypoperfusion volume, mL | 82.5(44.4-135.6) | 93.1(44.0-149.5) | 0.336 |
| Baseline mismatch ratio | 2.4(1.7-3.2) | 2.5(1.9-3.9） | 0.191 |
| CBF_hypo_, ml/min/100g | 5.7(4.6-8.5) | 7.3(5.2-9.1) | 0.047 |
| rPS_hypo_，% | 75.61(47.61-122.54) | 55.60(28.79-84.96) | 0.004 |
| HT, n (%) | 46(48.4) | 29(31.5) | 0.018 |
| HI, n (%) | 35(36.8) | 21(22.8) | 0.039 |
| PH, n (%) | 11(11.6) | 8(8.7) | 0.514 |

CBF_hypo_: cerebral blood flow in hypoperfusion region; HI: hemorrhagic infarction; HT: hemorrhagic transformation; NIHSS: national institute of health stroke scale; PH: parenchymal hematoma; rPS_hypo_: rPS in hypoperfusion region.
